# The Effects of Various Essential Oils on Epilepsy and Acute Seizure: A Systematic Review

**DOI:** 10.1155/2019/6216745

**Published:** 2019-05-22

**Authors:** Tyler A. Bahr, Damian Rodriguez, Cody Beaumont, Kathryn Allred

**Affiliations:** ^1^dōTERRA International, LLC, 389 S. 1300 W, Pleasant Grove, UT 84062, USA; ^2^University of Texas Health Science Center San Antonio, 7703 Floyd Curl Drive, San Antonio, TX 78229, USA

## Abstract

Many essential oils (EOs) have anticonvulsant activity and might benefit people with epilepsy. Lemongrass, lavender, clove, dill, and other EOs containing constituents such as asarone, carvone, citral, eugenol, or linalool are good candidates for evaluation as antiepileptic drugs. On the other hand, some EOs have convulsant effects and may trigger seizures in both epileptic and healthy individuals. Internal use of EOs like sage, hyssop, rosemary, camphor, pennyroyal, eucalyptus, cedar, thuja, and fennel can cause epileptic seizures because they contain thujone, 1,8-cineole, camphor, or pinocamphone, which have been identified as convulsive agents. While more research is needed to confirm their mechanisms of action, it appears that the convulsant or anticonvulsant properties of essential oils are largely due to (1) their ability to modulate the GABAergic system of neurotransmission and (2) their capacity to alter ionic currents through ion channels. This review presents a systematic analysis of the current research on EOs and epilepsy, including human case studies, animal models, and* in vitro* studies.

## 1. Introduction

Approximately 20-30% percent of patients with epilepsy suffer from seizures that cannot be controlled using any antiepileptic drugs (AEDs) that are currently available [1]. Although surgery is another method of controlling epilepsy, it is used selectively in patients whose seizures originate from a single, localizable site. Other treatments for epilepsy are still in developmental stages [1]. Although this generation's AEDs tend to have fewer side effects, there is still risk for psychological complications, such as anxiety, depression, and cognitive impairment [1]. Despite these limitations, AEDs are likely to remain the primary treatment for epileptic patients because of their ease of use and wide availability. According to Ekstein in 2010, “New drug therapies with efficacy against drug-resistant seizures, favorable adverse events profiles, especially in regard to neurological and psychiatric effects, and, if possible, low costs to patients and high worldwide availability are clearly needed” [2]. A large amount of evidence suggests that natural medicines may be one potential source of new antiepileptic drugs [2].

Essential oils (EOs) are one particular class of natural medicines obtained by distillation of plant material to obtain a volatile, hydrophobic extract. EOs have been used as anticonvulsants in traditional medicine in many cultures worldwide, especially in the Middle East, India, China, and Brazil. It is no surprise that much of the research on EOs and their antiepileptic effects has been produced by institutions in these regions. Even today, some herbal remedies are often more accessible than synthetic drugs for individuals in developing communities located in these parts of the world [3].

EOs have been documented for anxiolytic, sedative, neuroprotective, and anticonvulsive properties by academic research groups worldwide [4–7]. Despite a large amount of primary research describing the antiepileptic potential of many EOs, no prior review article has summarized these findings exclusively in the context of epilepsy and seizures to our knowledge.

Compounds found in EOs have been shown to interact with and exert pharmacological action on central nervous system targets involved in epilepsy. Structures involved in neurotransmitter release and metabolism such as NMDA, GABA_A_, GABA_B_, glycine, and acetylcholine receptors, as well as the acetylcholinesterase and GABA transaminase enzymes are modulated by certain EO compounds [8–14]. Other EO compounds influence neuronal excitability and action potential dynamics by modulating voltage-gated sodium and calcium channels [15, 16].

EOs and their constituent compounds have unique chemical properties that make them good candidates for drug design. Since the plant enzymes that produce terpenes are stereoselective, many EOs contain only one enantiomer of a compound. This can be advantageous in cases where one enantiomer has pronounced effects while the other is inactive or affects a different target. Another key property of EOs in the context of epilepsy is their ability to cross the blood-brain barrier (BBB). Drug candidates likely to cross the BBB are usually of small size (<400 Da) and high lipid solubility [17]. Virtually all compounds found in EOs meet these criteria.

## 2. Materials and Methods

### 2.1. Systematic Review

The present review employed a systematic search of the National Institute of Health PubMed database for all articles containing the keyword “essential oils,” together with either of the three keywords “epilepsy,” “epileptic,” or “seizure.” Only primary research articles published in the English language between 1900 and 2017 with relevant information on EOs and epilepsy were included in the review. Publications documenting the activity of methanolic or aqueous extracts but not EOs were excluded, as were articles describing the activity of EOs in the context of diseases other than epilepsy.

Of the 122 research articles identified in the initial search, fifty-eight were excluded. Of these fifty-eight, thirty-three did not contain information about essential oils in the context of epilepsy or seizures and were excluded for being irrelevant to the subject at hand. Eight were excluded because they were published in a language other than English, and sixteen others were excluded because they were review articles, commentaries, or other publications which are not considered original research. A total of sixty-four articles meeting the inclusion criteria were systematically reviewed and classified into two categories depending on whether the research documented positive or negative outcomes. Of the articles included in this review, fifty-four of the publications (84%) reported positive outcomes and ten of the publications (16%) reported negative outcomes.

### 2.2. Animal Models

Two main types of animal models emerged in this review: models of acute seizure and models of chronic epilepsy. Animal models of chronic epilepsy aim to simulate spontaneous seizure, neurological insult that results from seizure, and lasting changes in the epileptic brain. Animal models of acute seizure aim to simulate the hyperexcitation of neural circuitry that causes convulsions and neurological lesion.

The majority of the studies that investigated the effects of EOs on chronic epilepsy used the pilocarpine or electric kindling models. Pilocarpine is a muscarinic acetylcholine receptor agonist used to mimic complex human partial seizures. The pilocarpine model shares many similarities to human temporal lobe epilepsy in terms of neurochemistry and its effects on cerebral networks [81]. Drugs effective against complex partial seizures in humans are usually effective against spontaneous seizures in model animals chronically exposed to pilocarpine.

The two main models used to study acute seizure were pentylenetetrazole (PTZ) and maximal electroshock (MES) models. PTZ is thought to be a GABA_A_ receptor antagonist. Administration of PTZ triggers epileptiform activity that imitates absence seizures and generalized tonic-clonic seizures [81, 82]. Maximal electroshock is a method where electrodes are placed on the ears and an electric current sufficient to induce seizure is passed through the animal's central nervous system. MES is an animal model of tonic-clonic seizures and can also be used to study the changes in gene expression, cell signaling, and synaptic plasticity since there is no chemical agent acting on neurological structures after the seizure [81].

Some authors in the present review used other drugs to induce acute seizures. Above-threshold doses of pilocarpine and kainic acid, which are also used to model chronic epilepsy, can be effective models of acute seizure. Picrotoxin and strychnine are two other less common proconvulsants. Picrotoxin (PCTX) antagonistically binds to the GABA_A_ receptor complex and in theory works similarly to PTZ. Strychnine (STRN), a glycine and acetylcholine antagonist, can also be used to produce tonic-clonic, absence, and myoclonic seizures [81].

In the majority of the studies, EOs or their isolated compounds were administered via intraperitoneal injection and the dosage was standardized across all test animals by milliliters or milligrams per kilogram body weight. The animal studies included in this review were not screened for their inclusion or exclusion of controls.

### 2.3. Human Case Reports

No clinical trials have been conducted on EOs as antiepileptic drugs. Eight case studies of adverse events in humans were the only source of human clinical research data in this review. Generally, these were peer-reviewed publications produced by physicians or by researchers using data from hospital records.

## 3. Results

### 3.1. Essential Oils with Anticonvulsive Effects

Many EOs and their isolated constituents have been documented for their anticonvulsive properties. Oils high in monoterpenes and monoterpenoids such as a-pinene, limonene, myrcene, asarone, carvone, citral, eugenol, and linalool predominate. This data is consolidated in Table 1.

#### 3.1.1. Oils High in a-Pinene, Limonene, and Other Monoterpenes

EOs containing alpha-pinene and other monoterpenes show anticonvulsant effects in animal models.* Angelica archangelica*, which contains alpha-pinene, 3-carene, and limonene, decreases the duration of tonic convulsions and decreases the postseizure recovery time in PTZ mice [22]. It also increases latency for clonic convulsions and decreases mortality in PTZ seizures. The peel of* Citrus aurantium*, also high in limonene, increases latency to both PTZ- and MES-induced seizures, increases sleep time in pentobarbital-treated animals, and decreases anxiety [33].* Nigella sativa*, which contains alpha-pinene, p-cymene, and thymoquinone (a GABA_B_ agonist) [14] inhibits MES convulsions, but its activity can be reversed by GABA_A_ antagonists PCTX and bicuculline indicating a GABA_A_-dependent mechanism of action [40]. Both the myrcene and limonene chemotypes of* Lippia alba* increase the latency period and survival of mice with PTZ-induced seizures [56]. The alpha-pinene rich resin of* Gardenia lucida* prolongs pentobarbital hypnosis and protects against intensity and frequency of seizures and animal mortality in PTZ and MES models, with loss of motor function as a side effect [47].

#### 3.1.2. Oils High in Asarone

Asarone is a compound found in the rhizomes of plants of the genus* Acorus* and* Asarum*. Chronic daily treatment with the alpha isomer has been shown to improve the latency and severity of pilocarpine-induced seizures [18]. Coadministration of the alpha isomer with chlorpromazine decreased the effects of chlorpromazine in animals, suggesting competition for the same site [21]. Chlorpromazine is an antagonist of various receptors including dopamine, serotonin, histamine, adrenergic, and cholinergic receptors. In MES animals, EO of* Acorus tatarinowii* prolonged seizure latency and decreased the convulsive rate and mortality, although it had no effect on PTZ-induced seizures [19].* Acorus gramineus* EO was found to inhibit the GABA transaminase enzyme. Animals that inhaled the oil for 30 days while receiving regular PTZ treatments had significantly higher brain GABA levels and significantly lower glutamate levels than animals that did not inhale the oil. In fact, neurotransmitter levels in the animals that inhaled the oil during the PTZ seizure regimen were almost identical to the levels found in control animals that did not go through PTZ-induced seizures periodically [20].

#### 3.1.3. Oils High in Carvone

Carvone is a monoterpene ketone found in mint plants and some Mediterranean spices. The S (+) enantiomer is the primary chemical constituent of* Anethum graveolens* (dill) and* Carum carvi* (caraway) oils, while the R (+) enantiomer is the primary constituent of* Mentha spicata* (spearmint) oil and can also be found in some chemotypes of* Calamintha officinalis* (calamint) oil. Interestingly, the stereochemistry of carvone plays a major role in its anticonvulsant properties. In one study, 200 mg/kg of S-carvone significantly increased the latency of convulsions in animals treated with PTZ and PCTX while the same of dose of R-carvone had no effect [29].

*Carum carvi* oil, high in (S)-(+)-Carvone, effectively inhibits tonic-clonic seizures induced by PTZ without any neuromuscular side effects [28]. Spearmint oil has not been documented for any anticonvulsant effects. However, one study found the oil of* Calamintha officinalis* to moderately reduce the duration of convulsions and increase the latency period to PTZ seizures [27]. This effect might indicate a minor presence of S-carvone or perhaps the presence of active carvone derivatives. Epoxycarvone, for example, has antiepileptic effects on PTZ seizures in both its enantiomeric forms, however the (S)-isomer is more active [44]. Another carvone derivative, hydroxydihydrocarvone, increases seizure latency at high doses, but comes with negative side effects including palpebral ptosis, decreased response to touch, and decreased motor activity [48].

#### 3.1.4. Oils Hydrodistilled from* Cymbopogon spp*

EOs obtained from plants of the genus* Cymbopogon* contain multiple compounds with anticonvulsive activity including citral, citronellol, and citronellal.* Cymbopogon citratus* EO, primarily composed of citral, increases latency and decreases tonic convulsions in various models of acute seizure including the PTZ, MES, and STRN models [36, 38]. The effects of the EO were blocked by flumazenil and potentiated by diazepam, suggesting that citral modulates GABA neurotransmission [38]. Another study on* C. citratus* oil tested the effects of a similar dosage given orally rather than intraperitoneally, but found that the oral route of administration abolished its anticonvulsive effect [37].* Lippia alba* (citral chemotype), another EO high in citral, was shown to have anticonvulsive effects on PTZ mice [56].

*Cymbopogon winteranius* is primarily composed of citronellal.* C. winteranius* oil was not observed to affect STRN-induced seizures and had only moderate effects on PCTX seizures, but did significantly improve latency and number of convulsions in phenytoin, MES, and PTZ animals [31, 39]. Like* C. citratus, C. winteranius* oil also seems to exert anticonvulsant effects dependent on GABAergic transmission [38]. Citronellol, another compound found in* C. winteranius* and other* Cymbopogon* species, is nearly identical to citronellal except that it has an alcohol group rather than an aldehyde group. Isolated citronellol had significant anticonvulsive effects in animals [31]. A follow-up experiment investigated compound action potentials in nerve bundles. The amplitude of action potentials decreased more than 90% in a 6.4 mM solution of citronellol. Electrophysiology recordings revealed that citronellol had no effect on repolarization, but that it strongly depressed the initial depolarization during the action potential [31].

#### 3.1.5. Oils High in Eugenol

Like citronellol, eugenol also has a depressive action on action potentials. It activity as a sodium channel blocker has been confirmed using whole-cell electrophysiology. Isolated eugenol depressed transient and late components of the sodium current. It also decreased L-type calcium currents and delayed rectifier potassium currents at higher concentrations [16]. In animal models, treatment with eugenol decreased the duration and intensity of pilocarpine-induced seizures about threefold each and increased the latency by about 50%. Daily eugenol treatment in pilocarpine chronic epilepsy animals prevented neuronal loss in the hippocampus, decreased seizure stage, and decreased seizure mortality [46].

Eugenol is the primary constituent in* Eugenia caryophyllata* (clove) oil and some Mediterranean spice oils. One study on clove oil found that a dose of 0.1 mL/kg prevented all convulsions with a 100% survival rate in mice treated with MES protocol. This same dosage nearly doubled the threshold for PTZ-induced seizures, but produced some motor impairment [45].* Laurus nobilis* EO, also high in eugenol, prevented PTZ convulsions and 80% of MES convulsions at doses of 0.75 mL/kg and 1 mL/kg, respectively [49]. Eugenol is also the primary constituent in* Ocimum gratissimum*, which had modest anticonvulsant effects in MES and PTZ mice [59]. The presence of the proconvulsive constituent 1,8-cineole in this oil probably explains why this* O. gratissimum* showed weaker effects against seizures than other eugenol-containing EOs.

#### 3.1.6. Oils High in Linalool

Linalool is a monoterpene alcohol proven to potentiate GABA_A_ function in mammalian electrophysiology experiments [83]. Linalool derivatives and metabolites including linalool oxide, linalyl acetate, 8-oxolinalyl acetate, 8-carboxylinalyl acetate, and 8-oxolinalool also affect GABA_A_ function or have anticonvulsive effects [55, 83]. In snail neurons, linalool has an inhibitory effect on sodium channels and augments potassium currents. At lower concentrations, linalool suppresses spontaneous activity and PTZ-induced epileptiform activity. At higher concentrations, it somehow induces epileptiform activity which can be reversed by calcium channel blockers [52].

In addition, it is possible that linalool may have neuroprotective effects by modulating NMDA receptors. NMDA-mediated calcium toxicity is one major mechanism of injury from epileptic seizures. In vitro assays showed that linalool displaced an NMDA antagonist, MK801, which directly interacts with NMDA receptors [53]. This suggests a direct interaction between linalool and NMDA receptors; however, it is not currently known if this interaction results in NMDA receptor inhibition. Lavender EO and its main constituent linalool were also found to inhibit T-type calcium channels in human embryonic kidney cells [50]. If this mechanism applies to neurons, lavender might attenuate cellular excitability by decreasing intracellular calcium and might further protect against calcium toxicity during seizure events.

Lavender and other EOs high in linalool demonstrate strong anticonvulsive effects in animal models of seizure.* Zhumeria majdae*,* Cinnamosma madagascariensis*, and* Citrus aurantium* blossom oil all increased latency and survival and decreased convulsions in PTZ-treated animals [30, 32, 70]. In one experiment with lavender oil, inhalation of 1 mL of the EO vapor 15 minutes before PTZ treatment prevented all convulsions in 100% of the animals and resulted in a 100% survival rate. All animals in the control group (PTZ but no lavender oil) experienced seizures and there was a 100% mortality rate at this dose [51].

#### 3.1.7. Other Oils/Constituents with Anticonvulsive Activity

Many other EOs and their isolated constituents have demonstrated anticonvulsive activity in animal models. Cumin EOs of the species* Cuminum cyminum* and* Bunium persicum *have been shown to inhibit epileptiform activity in animal models and in* in vitro* neurons, respectively [25, 34]. Both share the same two major constituents, cuminaldehyde and p-cymene.

Certain sesquiterpene compounds have positive effects on animal models of epilepsy. Trans-caryophyllene has protective effects on kainic acid-induced seizure by inhibiting malondialdehyde synthesis and maintaining healthy catalase, superoxide dismutase, and glutathione peroxidase activity [26]. (+)-Dehydrofukinone, a sesquiterpene ketone, increases the latency to PTZ-induced seizures in mice. Whole-cell electrophysiology experiments showed that dehydrofukinone induced hyperpolarization, decreasing calcium mobilization from the synapse. Activity could be blocked by flumazenil, indicating that the compound's mechanism is GABAergic neuronal inhibition [41].* Smyrnium cordifolium* EO, which is high in the sesquiterpenes curzerene, cadinene, and elemene, protected PTZ mice against all convulsions and mortality at a dose of 0.4 mg/kg [35].

Monoterpene alcohols may have potential as AEDs. Terpineol prolonged narcotic effects of hexobarbital, ethyl alcohol, and chloral hydrate and protected against MES- and PTZ- but not STRN-induced convulsions [54]. Terpinen-4-ol was found to alleviate convulsions mediated by 3-MP (a GABA_A_ antagonist); however, its activity was not reversed by flumazenil, indicating that it does not bind to the benzodiazepine site [65]. It also protected against PTZ and PCTX-induced convulsions in mice [66]. In whole-cell electrophysiology experiments, terpinen-4-ol decreased the sodium current, so its anticonvulsant activity might involve an additional mechanism in which neuronal excitability is decreased [65]. In one study, peppermint oil, with its main constituent menthol, was tested against a panel of other EOs and was found to have the highest anticonvulsive activity.* Ocimum basilicum, Mentha spicata, Lavandula angustifolia, Rosmarinus officinalis, Mentha pulegium, Origanum dictamnus*, and* Origanum vulgare* were the other EOs tested [57].

Other monoterpenoids are documented for similar activity. The monoterpene ketone verbenone increased seizure latency more than tenfold and upregulated COX-2, BDNF and c-fos in PTZ animals [68].* Zataria multiflora* EO, high in monoterpene phenols carvacrol and thymol, increased the onset time of clonic seizures and prevented PTZ tonic convulsions in PTZ and MES mice [69]. SuHeXiang Wan oil, rich in borneol and eugenol, markedly delayed the appearance of PTZ-induced convulsions but showed only weak inhibition on PCTX- and STRN-induced convulsions. Daily inhalation of the oil inhibited the activity of GABA transaminase, increasing GABA content and decreasing glutamate content in the brain to levels similar to controls. The EO inhibited the binding of a GABA ligand at the benzodiazepine site [64].

Only a few EOs with nonterpene constituents have been found to inhibit seizures. These include* Myristica fragrans* (myristicin, elemicin, and safrole),* Dennettia tripetala* (1-nitro-2-phenylethane), and* Rosa damascena* (rose oxide and phenyl ethyl alcohol) [42, 58, 63].

### 3.2. Essential Oils with Proconvulsive Effects

Some EOs contain constituents with convulsant activity. Reports of adverse events in humans are the primary source of research on these EOs. EOs of the species* Salvia officinalis* (sage),* Thuja plicata* (thuja),* Cedrus spp.* (cedar),* Hyssopus officinalis* (hyssop),* Eucalyptus spp*. (eucalyptus),* Cinnamomum camphora* (camphor),* Mentha pulegium* (pennyroyal), and* Anethum graveolens* (fennel), as well as the constituents 1,8-cineole, camphor, thujone, and pinocamphone have produced seizures when used both internally and topically. Data on the EOs and constituents with convulsive properties are found in Table 2.

#### 3.2.1. Oils High in Thujone

Thujone is the primary chemical constituent in sage oil, although sage oil also contains significant levels of 1,8-cineole and camphor. The ingestion of small quantities of sage EO has caused tonic-clonic seizures in humans, especially in children [73, 76]. According to one report, a 33-day-old boy experienced tonic-clonic convulsions lasting 20 minutes after consuming an unknown amount of sage oil [78]. In another report, a 5-1/2-year-old girl ingested about 5 mL of sage oil and subsequently experienced a generalized tonic-clonic seizure lasting 10 minutes [78]. In another case report, a healthy 54-year-old woman with no history of epilepsy had taken sage orally for years as a purported treatment for her hyperlipidemia. One day, she took a higher dose than normal and noticed involuntary convulsions in her tongue. Half an hour later she experienced a generalized tonic-clonic seizure and then fell unconscious for a full hour (Burkhard et al., 1999). A 53-year-old man was given about ten drops of sage oil by a coworker and after 20 minutes he began to experience a tonic-clonic seizure followed by a coma that lasted fifteen minutes [73]. Animal studies investigated the minimum dosages necessary to produce convulsions and mortality. 0.5 g/kg was sufficient to trigger seizures and 3.2 g/kg was lethal [78].

Like sage oil, thuja and cedar EOs are high in thujone and are also known to cause convulsions, sometimes even when used topically [73, 76]. In one report, a 7-month-old child experienced eight tonic-clonic seizures at different times due to repeated topical exposure to an unknown amount of thuja EO. EEG and MRI scans appeared normal and seizures ceased after discontinuation of the thuja EO [79]. Toxicology studies on alpha- and beta-thujone revealed that a dosage of 25 mg/kg is sufficient to trigger seizures and a dosage of 50 mg/kg is lethal in 100% of mice [80].

#### 3.2.2. Oils High in 1,8-Cineole and Camphor

EOs of camphor, rosemary, and eucalyptus which are high in 1,8-cineole and camphor (note that camphor is the name of the EO* and* the isolated compound) have produced adverse epileptic reactions in humans. For example, in one report a 3-year-old girl of 15 kg had been using chest rub with 19% camphor oil for nasal congestion. Her father mistakenly gave her a teaspoon of the oil and within 20 minutes she had a generalized tonic-clonic seizure [74]. In another report, a 15-month-old boy of 10 kg opened a bottle of camphor oil and consumed 20 mL. He developed a generalized tonic-clonic seizure after 10 minutes [74]. In animal models, 1,8-cineole and camphor were both able to induce seizures at a dosage of 0.5 mL/kg [71].

Topical application of these EOs can also cause seizures, especially in people with epilepsy. Another patient with a history of epilepsy experienced a breakthrough (relapse) seizure after a massage with a blend of sea fennel, maritime pine, sea-buckthorn, and rosemary EOs. The camphor content in rosemary EO was thought to be the cause of the seizure. The patient had not had a seizure in 8 years and did not experience any seizures again for at least a year following the incident [72]. In another case report, a 12-month-old girl with no prior history of epilepsy was bathed in a wash containing an unknown quantity of eucalyptus, pine, and thyme oil. A few minutes after her last bath, she experienced an episode of tonic convulsions lasting about one minute and experienced two more similar episodes later that day. Over the next few days, her seizures became increasingly frequent and could not be controlled by anticonvulsant drugs. As a result of the seizures, the child was developmentally delayed and became prone to future seizures. The seizures persisted following the discontinuation of EO use; therefore the researchers suggested the child had underlying epileptogenic encephalopathy. Eventual epileptic events were likely for the child, but her exposure to EOs may have initiated and exacerbated the activity [73].

Paradoxically, oils high 1,8-cineole have produced some positive results in animal models of seizure.* Artemisia annua* EO, high in camphor, 1,8-cineole, and p-cymene, increased latency to pilocarpine and PCTX-induced convulsions and prevented onset of PTZ and STRN-induced seizures [23].* Ocimum gratissimum *EO, high in eugenol but also high in 1,8-cineole, showed good anticonvulsant activity as did the EO of* Zhumeria majdae*, which is high in linalool but also high in camphor [59, 70]. Elettaria cardamomum (cardamom) EO, which contains high levels of 1,8-cineole, significantly delayed the onset of clonic seizures, prevented all PTZ seizures, and prevented 62.5% of MES seizures at a dose of 1mL/kg [43]. Psidium guyanensis (guava) leaf EO, high in 1,8-cineole and also alpha-pinene, reduced the severity of PTZ but not STRN or PCTX seizures [62].* Tetrapleura tetraptera* EO, predominantly composed of 1,8-cineole, protected 78% of animals from leptazol-induced seizures at a dose of 0.4 mL [67]. One potential explanation for these conflicting results is that 1,8-cineole may be a weak partial GABA_A_ antagonist. It is possible that 1,8-cineole competes for the same site as other convulsant drugs; however, its effects are much weaker, giving the appearance of anticonvulsant activity.

#### 3.2.3. Other Oils with Proconvulsive Effects

Some other EOs that do not contain thujone, 1-8-cineole, or camphor can cause seizures. Hyssop EO, predominantly composed of the compound pinocamphone, can cause tonic-clonic convulsions in humans [73, 76]. In animals, 0.13g/kg was sufficient to trigger seizures and 1.25 g/kg was lethal [73]. Pennyroyal EO may cause epileptic and toxic effects due to its pulegone and menthofuran content, especially in infants [67, 78]. In one report, an infant consumed pennyroyal oil and was hospitalized for epileptic encephalopathy and liver failure. Blood levels of pulegone and menthofuran were 25 and 41 ng/mL, respectively [67].

*Anethum graveolens* (fennel) EO is also documented for having proconvulsive effects [73, 75]. A woman consumed a large number of cakes containing an unknown quantity of fennel EO and experienced tonic-clonic convulsions lasting 45 minutes. This is the only adverse report associated with fennel oil, and the chemotype of the fennel oil was unknown [75]. Fennel EO is usually rich in anethole; however, animal studies on oils high in anethole demonstrate that anethole likely possesses neuroprotective and anticonvulsant effects. For example,* Pimpinella anisum* EO elevated the PTZ seizure threshold and suppressed MES and PTZ convulsions in mice [60, 61]. It also inhibited the production of dark neurons in epileptic rats, a side effect of chronic epilepsy [61].* Artemisia dracunculus* oil, another oil high in anethole, showed dose-dependent anticonvulsive effects in both PTZ and MES mice [24]. Taken together, these results suggest that the adverse reaction associated with the fennel EO may not associated with anethole, but another chemical constituent found in fennel EO.

## 4. Discussion

### 4.1. Summary of the Research

In this review, we find that many EOs demonstrate anticonvulsant activity and might benefit people with epilepsy. Plants of the genus* Cymbopogon* or* Acorus* are likely to produce EOs with anticonvulsive properties. The chemical compounds asarone, carvone, eugenol, linalool, monoterpene alcohols, and some sesquiterpenes are also likely to have positive effects on epilepsy by acting on various nervous system targets.

Some of the research studies in this review described EOs that were composed of multiple constituent compounds, but many described EOs that were predominantly composed of one compound. When many oils containing the same major constituent had similar effects, it was inferred that that specific constituent was the active component responsible for the oils' anticonvulsive effects. However, further research is needed to confirm that these compounds are in fact the active components of the EOs.

### 4.2. Mechanisms of Action

While more research is needed to confirm their mechanisms of action, it appears that one mechanism for the anticonvulsant properties of EOs is their ability to modulate GABAergic neurotransmission. Alpha-asarone and SuHeXiang Wan oil, for instance, both inhibit the GABA transaminase enzyme, increasing brain GABA levels and decreasing brain glutamate levels in animal models of chronic epilepsy. The constituents linalool, alpha-pinene, thymoquinone, and terpinen-4-ol either potentiated GABA activity or were found to bind the GABA_A_ receptor at the benzodiazepine site.

A second mechanism explaining the anticonvulsive action of EOs is their capacity to block ionic currents. Eugenol, the principal component of clove oil, inhibits action potential generation by blocking sodium channels. Citronellol depresses the depolarization phase of action potentials in nerve fibers, probably by the same mechanism. Terpinen-4-ol also decreased sodium currents in electrophysiology experiments.

Blending is a popular practice among people who use EOs. Traditionally, mixtures and formulations of two or more EOs were believed to sometimes exhibit synergy. At this time however, no blends have been studied for their anticonvulsive potential except for SuHeXiang Wan oil, which did show significant effects on brain GABA levels in a model of chronic epilepsy. From these and other results discussed in this review, it is possible that a blend of anticonvulsive oils might serve as a multitarget pharmacological approach to controlling epilepsy and could be more effective than any single oil alone. For example, a combination of acorus EO high in asarone, lemongrass EO high in citronellol and citral, lavender EO high in linalool, and clove EO high in eugenol would simultaneously suppress action potentials, potentiate GABA_A_ receptor activity, and increase synaptic GABA levels by inhibiting the GABA transaminase enzyme. Future research should be conducted on antiepileptic blends.

### 4.3. CBD and Epilepsy

Another chemical that has recently become popular in the world of natural products and is undergoing investigation for possible benefits in regard to seizures and epilepsy is cannabidiol (CBD). CBD itself has been shown anecdotally and clinically to provide benefit and significant relief to epileptic patients [84, 85]. CBD comes from the* Cannabis* plant and is provided in many dosage forms, one of which is hemp essential oil. While present in hemp oil, CBD itself is not an essential oil; as such, studies which investigated CBD alone with regard to seizures and epilepsy do not fit in the scope of this review. As of the publication of this review, no primary research exists investigating hemp oil in the context of epilepsy.

### 4.4. Limitations

This review has limitations. The publications included in this review were gathered exclusively from PubMed; other scientific databases were not searched for relevant publications. Poison databases were not searched for reports of potential EO-induced seizures. Furthermore, the animal studies included in this review were not screened for their inclusion or exclusion of controls.

## 5. Conclusion

Because of their lipophilic nature, EO compounds can easily cross the blood-brain barrier. This property, combined with the aforementioned pharmacology of their constituents, makes EOs excellent candidates for investigation into their potential as AEDs. That said, certain EOs should be used with caution due to case reports and animal studies demonstrating that they may induce seizures, specifically EOs of sage, thuja, cedar, hyssop, eucalyptus, camphor, pennyroyal, and fennel, as well as the constituents 1,8-cineole, camphor, thujone, and pinocamphone. Future research will be necessary to determine the pharmacological action of these compounds, but GABA_A_ antagonism appears to be one potential mechanism.

Together, these results suggest that many EOs may be promising for treating people with epilepsy. While some EOs have convulsive properties, these observations cannot be generalized to all EOs. Many EOs have had positive effects on animal models of chronic and acute epilepsy. Because different EOs affect different targets, blends and formulations of EOs should be considered. Future experiments including human clinical trials should also be considered as a next step in verifying whether EOs might be used as AEDs in people with epilepsy.

## Figures and Tables

**Table 1:** Essential oils with anticonvulsant activity.

EO or Constituent	Study Type	Dosage	Effects	Reference
alpha-Asarone	animal (mice) PTZ, MES	200 mg/kg	Little effect on acute PTZ, MES model animals	[18]

alpha-Asarone	animal (rat) pilocarpine spontaneous recurrent seizures	200 mg / kg	Chronic daily treatment at this dose for 28 days abolished all convulsions and prevented mortality in 100% of animals. 100% of control animals experienced convulsions and mortality was 40% in the controls.	[18]

alpha-Asarone	animal (mice) MES	25 mg/kg	Protected against MES seizures. Interacted competitively with chlorpromazine.	[19]

beta-Asarone	animal (mice) MES	25 mg/kg	Slightly increased susceptibility and mortality. No effect on chlorpromazine activity.	[19]

*Acorus gramineus *rhizome	animal (mice) PTZ	30 days inhalation	Increased brain GABA levels and decreased glutamate content by inhalation of the oil. PTZ-seizure animals which inhaled the oil for 30 days had brain higher GABA levels and lower glutamate levels, close to the control animals which did not go through PTZ-induced seizures periodically. The mechanism was determined to be inhibition of the GABA transaminase enzyme.	[20]

*Acorus tatarinowii Schott* rhizome	animal (mice) PTZ, MES	1.25 g / kg;	ED50 for MES. No effect on PTZ induced seizures, but prolonged latency and decreased convulsive rate. Also decreased mortality.	[21]

*Angelica archangelica Linn.*	animal (mice) MES, PTZ	400 mg/kg	83% protection, 16% mortality from PTZ seizures	[22]

*Angelica archangelica Linn.*	animal (mice) MES, PTZ	500 mg/kg	100% protection from MES seizures, no mortality; duration reduced 20-fold, latency increased four-fold	[22]

*Artemisia annua L.*	animal (mice), PTZ, pilocarpine, PCTX, STRN	470 mg/kg	ED50 for PTZ seizures. Increased latency to pilocarpine and PCTX-induced convulsions. Prevented onset of PTZ and STRN-induced seizures. Motor inhibition was a side effect.	[23]

*Artemisia dracunculus L.*	animal (mice) PTZ, MES model	0.84 mL / kg	ED50 for MES animals	[24]

*Artemisia dracunculus L.*	animal (mice) PTZ, MES	0.26 mL/kg	ED50 for PTZ animals	[24]

*Bunium persicum*	animal (mice) PTZ, MES	1 mL / kg	0% of convulsive movements compared to PTZ-only control	[25]

*Bunium persicum*	animal (mice) PTZ, MES	1.25 mL / kg	0% of convulsive movements compared to MES only control	[25]

trans-Caryophyllene	animal (mice) kainic acid	60 mg/kg	Reduced mortality by 50% compared to kainic acid-only group. Significantly reduced seizure activity score around two-fold. Also lessened seizure severity by inhibiting malondialdehyde synthesis and preserving activity of GPx, SOD, and CAT. Reduced levels of the inflammatory cytokines TNF-a and IL-1B.	[26]

*Calamintha officinalis*	animal (mice) PTZ	50 mg/ kg	55% reduction in average duration of convulsions, latency period 21.7 times longer than controls and comparable to animals treated with 1 mg/kg diazepam.	[27]

*Calamintha officinalis*	animal (mice) PTZ	100 mg/kg	75% reduction in seizure duration, latency period 22.2 times longer than controls	[27]

*Carum Carvi L. *	animal (mice) PTZ	42.3 mg/kg	ED50 for PTZ clonic seizures	[28]

*Carum Carvi L.*	animal (mice) PTZ	97.6 mg/kg	ED50 for PTZ tonic seizure	[28]

R-Carvone	animal (mice) PTZ, PCTX	200 mg/kg	no effect	[29]

S-Carvone	animal (mice) PTZ, PCTX	200 mg/kg	Significantly increased latency of convulsions	[29]

*Cinnamosma madagascariensis Danguy*	animal (rats) PTZ	0.8 mL/kg	Prevention of all convulsions and mortality. Some slight sedative effects were observed.	[30]

Citronellol	animal (mice) PTZ, MES, PCTX	400 mg / kg	Increased seizure latency by around 50% and reduced the percent of animals with convulsions by 75% in PTZ model. For MES animals, the reduction in convulsions was identical at the same dosage of 400 mg/kg, with 75% protection from tonic convulsions.	[31]

Citronellol	In vitro nerve fibers	6.4 mM solution	Compound action potentials reduced by 90% in nerve bundle bathed in citronellol. There was no effect on repolarization, but only the initial depolarization.	[31]

*Citrus aurantium *blossom	animal (mice) PTZ, MES	40 mg / kg	Increased the clonic seizure threshold by 50%. The EO provided 92% seizure protection and 100% survival, compared to 0% protection and 30% surivival in controls. flumazenil reversed protection, indicating the involvement of GABA-ergic system.	[32]

*Citrus aurantium* peel	animal (mice) MES, PTZ	1g/kg	Increased latency period for MES and PTZ	[33]

*Cuminum cyminum Linn*	in vitro neurons, PTZ	1% v/v	Decreased spontaneous activity induced by PTZ in a concentration dependent manner	[34]

Curzerene	animal (mice) PTZ	0.4 mg/kg	100% prevention of PTZ convulsions and mortality	[35]

Curzerene	animal (mice) PTZ	0.25 mg/kg	ED50	[35]

*Cymbopogon citratus*	animal (mice) MES, PTZ	1 g/kg	Delayed clonic seizures induced by PTZ and blocked tonic extensions induced by MES. Prevented 40% of tonic convulsions in PTZ animals and 80% of tonic convuslions in MES animals. No significant effect on clonic convulsions.	[36]

*Cymbopogon citratus*	animal (mice) PTZ	oral dose of 200 mg/kg	No effect	[37]

*Cymbopogon citratus* & *Cymbopogon winteranius*	Animal (mice) PTZ, STRN	200 mg/kg	Increased seizure latency 8-fold and also increased latency to death in both PTZ and strychinine models. Effects blocked by flumazenil and potentiated by diazepam.	[38]

*Cymbopogon winterianus*	animal (mouse) PTZ, PCTX, phenytoin, STRN	200 mg/kg	Seizure latency increased nearly seven fold. Percent of animals experiencing convulsions was reduced by 50% and survival increased from 20% (control) to 70% (EO treatment group).	[39]

p-Cymene	animal (mice) MES	970 mg/kg	ED50 for MES seizures	[40]

p-Cymene	animal (mice) PTZ	393 mg/kg	ED50 for PTZ seizures	[40]

Dehydrofukinone	in vitro and animal (mice) PTZ	100 mg/kg	Delayed onset of generalized tonic-clonic seizures. Induced hyperpolarization of neurons via GABA activation. Decreased calcium mobilization from synapse. Activity could be reversed by flumazenil.	[41]

*Denettia tripetala*	animal (mice) PTZ, STRN	200 mg/kg	100% protection from PTZ and STRN-induced convulsions. Co-treatemnt with flumazenil, a GABA receptor antagonist, abolished the anticonvulsant effects on the EO and the constituent.	[42]

*Elettaria cardamomum*	animal (mice) PTZ, MES	1 mL/kg	Significantly delayed onset of clonic seizures, prevented all PTZ seizures and 62.5% of MES seizures at this dose. Showed some degree of movement toxicity.	[43]

(-)-Epoxycarvone	animal (mice) PTZ, pilocarpine, STRN	300 mg/kg	Only 12.5% inhibition of PTZ convulsions. No effect on STRN animals. Protected against pilocarpine seizures.	[44]

(+)-Epoxycarvone	animal (mice) PTZ, pilocarpine, STRN	300 mg/kg	Increased latency to PTZ-induced seizure onset with 100% survival. Prevented tonic seizures induced by MES. Exhibited 25% inhibition of PTZ convulsions. No effect on strychine animals. Protected against pilocarpine seizures.	[44]

*Eugenia caryophyllata*	animal (mice) MES, PTZ	0.1 mL/kg	Abolished all convulsions in MES mice and 100% survival. Nearly doubled PTZ seizure threshold, but only reduced convulsions by 20% in mice above the threshold.	[45]

Eugenol	animal (mice) pilocarpine	-	No difference in seizure latency, but decreased duration and intensity of pilocarpine-induced seizures about threefold each.	[16]

Eugenol	patch-clamp electrophysiology	-	Depressed transient and late components of sodium current. It also decreased L-type calcium currents and delayed rectifier potassium currents at higher concentrations.	[16]

Eugenol	animal (rats) pilocarpine	100 mg/kg for 7 days	55% reduction in average duration of convulsions. Latency period was 21.7 times longer than controls and comparable to animals treated with 1 mg/kg diazepam. Neuronal loss was prevented by eugenol treatment in epileptic animals in all hippocampal sub-regions including DG, CA3, and CA1. Seizure stage and mortaility were improved.	[46]

*Gardenia lucida* resin	animal (mice) MES, PTZ	300 mg/kg	For MES animals, the EO reduced convulsion time nearly tenfold and reduced recovery time six-fold. In PTZ animals, the EO increased latency fourfold and reduced number of convulsions twofold. Loss of motor function was a side effect.	[47]

Hydroxydihydrocarvone	animal (PTZ)	400 mg/kg	PTZ seizure latency increased two-fold. Side effects included palpebral ptosis, decreased response to touch, increased sedation. Decreased motor activity. Protected against PTZ-induced convulsions.	[48]

*Laurus nobilis* leaf	animal (mice) PTZ, MES	0.75 mL/kg	Prevented all convulsions in PTZ mice and 0% mortality. Also produced sedation and motor impairment at anticonvulsant doses	[49]

*Laurus nobilis* leaf	animal (mice) PTZ, MES	1 mL/kg	In MES animals, prevented 80% of convulsions. Only 10% mortality. Also produced sedation and motor impairment at anticonvulsant doses.	[49]

*Lavandula angustifolia*	in vitro human embryonic kidney cells	0.034 mg/mL	Lavender and rosemary essential oils both inhibit CaV3.2 T-type calcium channels. Linalool was determined to be the active component.	[50]

*Lavandula angustifolia*	animal (mice) PTZ, strychinine	Inhalation of 1 mL	Inhalation of 1 mL of lavender oil 15 minutes before treatment with 50 mg/kg PTZ prevented all convulsions in 100% of the animals and prevented mortality. All animals in the control group experienced seizures and there was a 100% mortality rate at this dose. In this experiment, lavender had no effect on STRN induced seizures.	[51]

Linalool	in vitro snail neurons	0.1 mM	supressed spontaneous activity and PTZ induced epileptiform activity	[52]

Linalool	in vitro snail neurons	0.4 mM	Induced epileptiform activity. This epileptiform was reversed by calcium channel blockers.	[52]

Linalool	in vitro	-	In vitro assays showed that linalool displaced an NMDA antagonist, MK801, which directly interacts with NMDA receptors. This suggests a direct interaction between linalool and NMDA receptors. There was no effect on muscimol binding, so no conclusive evidence was obtained about a GABAergic mechanism.	[53]

Linalool	animal (mice) MES, PTZ, STRN	-	Increased latency period and decreased mortality in all models	[54]

Linalool oxide	animal (mice) MES, PTZ	150 mg/kg	Moderately reduced duration of tonic seizures induced by MES and increased latency to PTZ seizures.	[55]

*Lippia alba, *citral chemotype	animal (mice) PTZ	100 mg/kg	Increased seizure latency and percentage of survival	[56]

*Lippia alba,* limonene chemotype	animal (mice) PTZ	200 mg/kg	Increased seizure latency and percentage of survival	[56]

*Lippia alba, *myrcene chemotype	animal (mice) PTZ	200 mg/kg	Increased seizure latency and percentage of survival	[56]

*Mentha piperita*	animal (mice) PTZ	1.6 mL/kg	Completely prevented all seizures at all and produced a rate of 100% survival.	[57]

*Mentha spicata*	animal (mice) PTZ	1.6 mL/kg	12-fold increase in seizure latency	[57]

*Myristica fragrans*	animal (mice) MES, STRN, bicuculline, PTZ	0.2 mL/kg	Increased latency to PTZ seizure and death 2-fold. 100% protection from convulsions induced by MES. Delayed onset of convuslions by STRN. At high doses, was a weak proconvulsant. No motor impariment was observed.	[58]

*Ocimum gratissimum L.*	animal (mice) MES, PTZ	1g/kg	Average of about 30 percent protection from MES convulsions. Little effect on PTZ convulsions	[59]

*Pimpinella anisum* fruit	animal (mice) MES, PTZ	1 mL/kg	Nearly doubled the PTZ seizure threshold. Protected against 80% of convulsions and prevented death in 90% of animals for both PTZ and MES conditions.	[60]

*Pimpinella anisum* fruit	animal (mice) PTZ	3 mL / kg	Latency increased five-fold with a treatment of 3 mL / kg. Inhibited production of dark neurons in different regions of brain in epileptic rats. Prolonged latency and reduced amplitude and duration of PTZ seizures.	[61]

alpha-Pinene	animal (mice) PTZ	440 mg/kg	ED50 for PTZ seizures	[40]

*Psidium Guyanensis *leaf	animal (mice) PTZ, PCTX, STRN	400 mg/kg	Reduced severity of PTZ seizures but not strychine or picroptoxin. Caffeine reversed the effect, suggesting that the mechanism involves the adenosine system.	[62]

*Rosa damascena*	animal (amygdala electrical kindling)	750 mg/kg	Number of stimulations necessary for first appearance of seizure was larger in animals treated with the EO. Seizure duration was shorter in the treatment groups.	[63]

*Rosmarinus officinalis*	in vitro human embryonic kidney (HEK) cells	0.054 mg/mL	Rosemary essential oil was found to inhibit CaV3.2 T-type calcium channels. Rosmarinic acid was found to be the active component.	[50]

*Smyrnium cordifolium*	animal (mice) PTZ	223 mg/kg	ED50	[35]

SuHeXiang Wan	animal (mice) PTZ	Inhalation for 3 hrs at a time, twice per day	3 hr inhalation twice per day doubled onset latency of PTZ-induced seizures and abolished lethality. Effects were minimal for pcrotoxin and strychinine treated animals. Inhalation of the oil inhibited the activity of GABA transaminase, increasing GABA content and decreasing glutamate content in the brain to levels similar to controls. EO inhibited the binding of a GABA ligand at the benzodiazepine site.	[64]

Terpinen-4-ol	animal (mice) PTZ	200 mg/kg	Increased latency period to PTZ-induced seizure 10 fold and latency to 2-MP induced seizure 5-fold, with activity comparable to 4 mg/kg DZP in both cases. Prevented 87% of seizures induced by PTZ. Alleviated 3-MP (a gaba antagonist) mediated convulsions. However, flumazenil didn't reverse the effect. Decreased I_Na through voltage-dependent sodium channels.	[65]

Terpinen-4-ol	animal (mouse) MES, PTZ, PCTX	200 mg/kg	Significantly increased latency of convulsions and inhibited PCTX induced seizures	[66]

Terpinen-4-ol	animal (mouse) MES, PTZ, PCTX	300 mg/kg	Decreased tonic convulsions at 300 mg/kg.	[66]

Terpineol	animal (mice) MES, PTZ, STRN	-	Increased latency period and decreased mortality in all models	[54]

*Tetrapleura tetraptera*	animal (mice) leptazol	0.4 mL	Protected 78% of animals at a dose of 0.4 mL.	[67]

Thymoquinone	animal (mice) PTZ	93 mg/kg	ED50 for PTZ seizures	[40]

1S-(-)-Verbenone	animal (mice) PTZ	200 mg/kg	Increased seizure latency more than ten-fold. Upregulated COX-2, BDNF and c-fos.	[68]

*Zataria Multiflora*	animal (mice) PTZ, MES	0.35 mL/kg	Significantly increased latency period for tonic convulsions and completely prevented tonic convulsions.	[69]

*Zhumeria majdae*	animal (mice) MES, PTZ	0.26 mL/kg	ED50 for PTZ and MES induced convulsions	[70]

**Table 2:** Essential oils with proconvulsive activity.

EO or Constituent	Study Type	Dosage	Effects	Reference
1,8-Cineole (isolated constituent)	animal	0.5 mL/kg	Induced tonic-clonic seizures	[71]

Blend (rosemary EO and camphor constituent)	human adult man	unknown, applied topically	Breakthrough (relapse) seizure in an epileptic patient after 8 years free of seizures	[72]

Blend (eucalyptus, pine, and thyme EOs)	human (12 months)	unknown, applied topically	Three episodes of tonic convulsions lasting one minute each. Hundreds of similar seizures the next day. As a result, the patient developed long-term status epilepticus and showed developmental delay for at least 4 years following the event.	[73]

Camphor (isolated constituent)	animal	0.5 mL/kg	Induced tonic-clonic seizures	[71]

Camphor oil	human (3 years)	about 1 teaspoon taken internally	Generalized tonic-clonic seizure and respiratory depression within 20 minutes	[74]

Camphor oil	human (15 months)	about 20 mL	Generalized tonic-clonic seizure after 10 minutes	[74]

Fennel oil	human adult woman	unknown but large amount	Tonic-clonic seizure lasting 45 minutes	[75]

Hyssop oil	animal	0.13 g/kg; 1.25 g/kg	Caused convulsions; lethal dose	[76]

Pennyroyal oil	human infant	25 ng/mL blood pulegone content and 41 ng/mL blood menthofuran content	Epileptic encephalopathy in and liver failure	[77]

Sage oil	animal	0.5g/kg; 3.2 g/kg intraperitoneally	Caused convulsions; lethal dose	[76]

Sage oil	human (53 yrs)	10 drops taken internally	Tonic-clonic seizure followed by 15-minute coma	[73]

Sage oil	human (54 yrs)	mouthful-sized amount taken internally	Tonic-clonic seizure, unconscious for 1/2 hour following the seizure	[73]

Sage oil	human (33 days)	unknown, taken internally	33-day old boy experienced tonic-clonic convulsions lasting 20 minutes	[78]

Sage oil	human (5 1/2 yrs)	5 mL taken internally	Generalized tonic-clonic seizure lasting 10 minutes	[78]

Sage, cedar, thuja, hyssop	human (multiple cases)	unknown, taken internally	Tonic-clonic convulsions in humans	[76]

Thuja (arborvitae) oil	human (7-months)	unknown, applied topically	8 tonic-clonic seizures at different times	[79]

Thujone (isolated constituent)	animal	25 mg/kg; 50 mg/kg	All animals experienced seizures; all animals died	[80]
